# Effects of Sleep Deprivation on Blood Glucose, Food Cravings, and Affect in a Non-Diabetic: An N-of-1 Randomized Pilot Study

**DOI:** 10.3390/healthcare8010006

**Published:** 2019-12-25

**Authors:** Eric Jay Daza, Katarzyna Wac, Marily Oppezzo

**Affiliations:** 1 Stanford Prevention Research Center, Stanford Medicine, Stanford, CA 94305, USA; 2 Clarify Health Solutions, San Francisco, CA 94105, USA; 3 Quality of Life Technologies Lab, University of Copenhagen, 1165 Copenhagen, Denmark; 4 Quality of Life Technologies Lab, University of Geneva, 1205 Geneva, Switzerland

**Keywords:** sleep deprivation, blood glucose, mood, food craving, calorie consumption, n-of-1 trial, self-experimentation, autoexperimentation

## Abstract

Sleep deprivation is a prevalent and rising health concern, one with known effects on blood glucose (BG) levels, mood, and calorie consumption. However, the mechanisms by which sleep deprivation affects calorie consumption (e.g., measured via self-reported types of craved food) are unclear, and may be highly idiographic (i.e., individual-specific). Single-case or “n-of-1” randomized trials (N1RT) are useful in exploring such effects by exposing each subject to both sleep deprivation and baseline conditions, thereby characterizing effects specific to that individual. We had two objectives: (1) To test and generate individual-specific N1RT hypotheses of the effects of sleep deprivation on next-day BG level, mood, and food cravings in two non-diabetic individuals; (2) To refine and guide a future n-of-1 study design for testing and generating such idiographic hypotheses for personalized management of sleep behavior in particular, and for chronic health conditions more broadly. We initially did not find evidence for idiographic effects of sleep deprivation, but better-refined post hoc findings indicate that sleep deprivation may have increased BG fluctuations, cravings, and negative emotions. We also introduce an application of mixed-effects models and pancit plots to assess idiographic effects over time.

## 1. Introduction

### 1.1. Background

The recent explosion of self-tracking wearable devices used for data collection and self-knowledge represents a “new culture of personal data” [1]. People can track more behaviors (e.g., sleep, activity, mood) with less labor (e.g., written food diaries), and can use these personalized data to hack their way into self-optimization for personalized health. At its simplest, consumers can monitor a single behavior and use their self-tracking data to modify it in desired directions, like walking more steps or eating a target number of calories.

The spread of technological tracking across health behaviors means that such tracking can be combined with disciplined scientific approaches in a more integrated framework to provide more robust personalized insights. Combining n-of-1 randomized trial (N1RT) study design with integration across time-stamped data, we can establish a more rigorous template for collecting and using self-quantification to inform individualized health plans [2,3]. Further, using a biometric tracker like continuous glucose monitoring adds a disease-prevention component to the personalized plan.

This study deploys an n-of-1 randomized trial (i.e., a randomized cross-over longitudinal or time-series study) and integrates data from three technological touchpoints: a consumer-grade tracking device, a continuous glucose monitoring (CGM) device, and an electronic survey. We use a subset of the data to explore the impact of sleep deprivation, an important health concern, on subsequent glucose control, food cravings, and mood in a non-diabetic individual. This study is distinctly not a case report, i.e., it is not a purely observational study of a participant, but rather includes randomized controlled trial (RCT) design elements that improve the validity of findings beyond what a case report can achieve [4]. In this way, an N1RT enables replicability of findings for a given individual that is hard to achieve with standard group-based RCTs [5].

The objective of this paper is twofold. The first is to establish the feasibility of n-of-1 randomized trials to characterize the idiographic (i.e., individual-specific) impact of a treatment (i.e., sleep deprivation) on a single subject’s self-tracked outcome. With abundant self-trackers and opportunities for individuals to track personal responses to behavioral changes, guidance in systematically testing and exploring this kind of data through an n-of-1 trial is not as widely recognized or utilized. Therefore, we demonstrate a more experimentally rigorous study design and analysis plan to contribute to and help guide self-tracking and n-of-1 trial design. Our effort is aligned with and complements seminal resources in n-of-1 biomedical and clinical study design such as Duan et al. [6] and Nikles and Mitchell [5].

The second objective is to inform future n-of-1 study design by instantiating this novel application of a rigorous personalized design within an exploratory investigation of the effects of sleep deprivation on glucose levels, cravings, and mood (representing physical, physiological, and psychological outcomes). We chose sleep as the intervention variable because it has such a profound and acute impact on several aspects of health and is a physical behavior that is commonly tracked on current consumer devices. Circulating glucose level is our selected health biomarker because it is the primary outcome measured for disease management in diabetics, and is also affected by sleep in non-diabetic individuals [7]. We chose cravings and moods as psychological outcomes (tracked electronically throughout the day) because these are commonly influenced by sleep deprivation. This hypothesis exploration is based on the extant literature on sleep deprivation’s effects on health, and the purposive choice of non-diabetic individual was with an eye for prevention of a chronic disease involving glucose metabolism.

### 1.2. N-of-1 Design

N-of-1 studies are scientific study designs focused on one individual, and are also known as single-subject or single-case studies. These designs are used extensively in the fields of psychology and education, and are increasingly used in clinical trials and biomedical research [8,9,10,11,12].

In a typical n-of-1 randomized trial, the study participant acts as their own baseline (i.e., control) via a randomized crossover design, e.g., ABAB, where A and B represent an active and baseline treatment, respectively. In this way, N1RTs provide bespoke evidence for making treatment decisions most relevant to a specific individual rather than an “average” individual from the target population [5,13].

### 1.3. Sleep Deprivation, Glucose, Cravings, and Affect

Within the last decade, sleep has emerged into the health spotlight as a crucial element for health. Sleep has been defined as “a reversible behavioral state of perceptual disengagement from and unresponsiveness to the environment … a complex amalgam of physiologic and behavioral processes.” [14]. The American Academy of Sleep Medicine and Sleep Research Society have developed a recommendation for sleep, and noted several characteristics of healthy sleep. These included the absence of sleep disorders, and the regularity, quality, and quantity of sleep, with quantity being the most common measure [15]. We used sleep duration in our study, as it is the most commonly researched, able to be objectively recorded by the device, and the easiest to manipulate by restricting sleep as the trial intervention.

Sleep plays an important role in glucose metabolism, appetite, endocrine, and immune function [16]. Chronic inadequate sleep is associated with obesity, insulin resistance, type 2 diabetes, metabolic syndrome, and cardiovascular diseases. Acute sleep deprivation, even a single night of it, has immediate cognitive, physical, physiological, and psychological effects. This is not just for those with extant glucose metabolic disorders, but also for non-diabetic individuals [7,16,17,18,19,20].

Sleep deprivation can affect glucose levels both directly and obliquely. From a biological standpoint, sleep deprivation causes metabolic dysregulation through myriad pathways involving sympathetic overstimulation, hormonal imbalance (implicating leptin, ghrelin, PYY, glucose, insulin, glucocorticoids, catecholamines, fatty acids, triglycerides), and subclinical inflammation (IL-6, TNF-α, and CRP) [7]. Consequently, lack of sleep has been shown to influence caloric consumption [15,19,20,21] and cravings for sweet or salty foods [22]. At the level of glucose homeostasis, sleep deprivation can decrease insulin sensitivity in healthy individuals without adequately compensating beta-cell function, which can lead to temporary impaired glucose tolerance [21].

Neurologically, compared to a sleep-rested state, sleep deprivation diminishes activity in areas of the brain associated with food choice (hypothetically leading to poor choices), and amplifies activity in an area known to signal food salience and promote eating. Behaviorally, some studies indicate that there is increased intake in fat [22] or in both fat and carbohydrates in sleep-deprived individuals [23]. The reason for including self-reports on cravings in this study is that cravings for sweet foods may lead to their increased intake; hence, if glucose control is altered, this could lead to an increase in glucose levels.

Finally, sleep deprivation has been shown to influence emotions [24]. Poor sleep quality correlates with high negative and low positive emotions [24], and there is evidence for an increase in anxiety after sleep deprivation [25,26]. Indeed, the psychological consequences of sleep loss are viewed as severe, to the extent that prolonged sleep deprivation has been regarded as torture when used in imprisonment contexts; sleep deprivation is even prohibited by the Physicians for Human Rights report [27]. Positive affect is also affected, with subjective decreased positive emotions in one night of sleep deprivation [28].

The rest of our paper is organized as follows. We present our three study hypotheses, the study design, and our analysis plan in the Materials and Methods section; in particular, how to collect and analyze data across devices and time within one individual using an N1RT. We report our main findings in the Results section, along with post hoc hypotheses and findings that can better inform a future N1RT design. We summarize our findings in the Discussion section, and reflect on our findings and experiences in conducting this pilot study in the Conclusions section.

## 2. Materials and Methods

### 2.1. A Priori Hypotheses

We investigated the following three a priori hypotheses regarding non-diabetics. Hypothesis 1 (H1) was that sleep deprivation, defined as restricting sleep to four hours per night, decreases homeostatic blood sugar control the next day. We expected to observe elevated glucose levels for longer intervals of time on average on post-sleep-deprivation days (i.e., day that follows a night of sleep deprivation) compared with baseline days (i.e., day that follows a regular night sleep without sleep deprivation). Hypothesis 2 (H2) was that sleep deprivation induces higher craving for high energy foods the next day. We expected to observe more cravings for high energy foods on post-sleep-deprivation days compared with baseline days. Hypothesis 3 (H3) was that sleep deprivation increases negative emotions and decreases positive emotions the next day. We expected to observe more negative emotions and less positive ones on post-sleep-deprivation days compared with baseline days.

### 2.2. Study Design

This study originally consisted of two N1RTs. Authors EJD and KW were the study participants and were randomized to eight periods during a 24 days study period.

Each N1RT was comprised of eight treatment periods (hereafter, “periods”), with four periods at each of two treatment levels. Randomization would serve to reduce confounding of the treatment effect for a given period. This would allow us to better assess up to four other factors that could affect an N1RT outcome: autocorrelation with past outcomes, a time trend in the outcomes (e.g., due to unobserved causes), carryover of treatment effects from past periods, and the trajectory of treatment effects during a period.

The three outcomes were defined as follows.

- Blood glucose (BG) level, a continuous variable, was measured in units of mg/dL every 15 min on average (SD = 8.6 s; median = 13.59 min, IQR = 19 s) using a continuous glucose monitoring (CGM) device.
- Craving was defined as an intense desire to consume a particular food or food type that is difficult to resist. The participant was asked whether they felt a craving for a particular food and would answer “yes” or “no”; if they had just eaten, they were to answer the question for craving felt before they ate.
- Participant affect was assessed using the Positive and Negative Affect Scale (PANAS) [29], a list of 20 feeling/emotion words that the participant rated on a 5-point scale (i.e., not at all, a little, moderately, quite a bit, extremely). Cravings were scheduled to be measured at the start (as a morning participant-initiated “pull” around 08:00 or as soon as the participant woke up), middle (midday until around 15:00), and end (evening around 20:00) of each 24-h study day. Affect was scheduled to be measured at the start and end of each 24-h study day.

Treatments and periods were defined as follows.

- Sleep deprivation was defined as getting at most four hours of sleep in one night.
- Active treatment was defined as sleep deprivation on the first night preceding a three-day period, followed by a two-night sub-period for recovery to “wash out” the effects of sleep deprivation (i.e., the “washout sub-period”).
- Baseline treatment was defined as getting at least six hours of sleep per night on all three nights of a period, with the last two nights mimicking an active-treatment washout sub-period. The latter pseudo washout sub-periods were included to try to avoid any effects of assigning irregular treatment periods (e.g., stress over uncertainty in personal scheduling).
- Active-treatment periods were designated as treatment level “A”, while baseline-treatment periods were designated as “B”. We were interested in treatment effects during the first day of each period, defined as the sleep-deprivation effects during the 24-h day immediately following a night of sleep deprivation starting at 06:00. We called this first day the “effect sub-period”.
- Recovery was defined as getting at least 14 h of accumulated sleep during the washout sub-period, with at least six hours on one of the nights, in order to fully recover from sleep deprivation (i.e., for outcomes to return to average baseline levels). To try to minimize the effect of unassigned sleep deprivation carrying over into a subsequent period, it was important to enforce at least six hours of sleep per night on the recovery nights. For example, suppose we only require 14 h of total sleep during both recovery nights. If a participant split their recovery sub-period as 10 h the first night and 4 h the next, they would have been sleep deprived on the second washout night. We allowed for it to take at least two nights of at least six hours of sleep each to recover from sleep deprivation. Hence, this non-randomized night of sleep deprivation might affect the outcome during day 1 of the following period, thereby worsening the observed day-1 effects in that period.

The self-reported outcomes were recorded using Qualtrics (Provo, UT, USA), a web-based survey instrument. CGM data in units of mg/dL were recorded using a Freestyle Libre (Abbott Diabetes Care Inc., Alameda, CA, USA) device.

### 2.3. Sleep Duration and Deprivation Monitored with Fitbit

Fitbit Charge 2™ (Fitbit, Inc., San Francisco, CA, USA) is a wearable fitness-tracker wristband able to track daily activity levels and sleep. The device automatically connects via Bluetooth and transfers data to a mobile platform via the Fitbit app. Fitbit Charge 2™ allows tracking of sleep stages (i.e., minutes spent awake, in “light”, “deep”, and rapid eye movement [REM] sleep) in addition to sleep/wake states; however, we did not assess sleep stages in this study.

As disclosed on fitbit.com, “Fitbit estimates your sleep stages using a combination of your movement and heart-rate patterns. Additional data—such as the length of time your movements are indicative of sleep behavior (such as rolling over, etc.)—help confirm that you’re asleep. While you’re sleeping, your device tracks the beat-to-beat changes in your heart rate, known as heart rate variability (HRV), which fluctuate as you transition between light sleep, deep sleep, and REM sleep stages. When you sync your tracker in the morning, we use your movement and heart rate patterns to estimate your sleep cycles from the previous night.”

For validation, de Zambotti et al. [30] compared Fitbit Charge 2™ to polysomnography, and found statistically apparent (i.e., statistically significant at the 0.05 level, or *p* < 0.05) evidence that Fitbit Charge 2™ overestimated polysomnography total sleep time by 9 min on average. For our purposes, this upward bias of 9 min was negligible: We could still use the device to check that KW complied with her assigned sleep-deprivation treatments, as we were interested in total sleep time on the scale of hours, not minutes.

### 2.4. Randomization Plan and Study Period

We implemented the following non-blinded, randomized-block design. A treatment block (hereafter, “block”) was defined as two consecutive three-day periods, with one A period and one B period. On the first study day, the participant was randomized (i.e., randomly assigned) either to block AB (i.e., treatment A followed by B) or block BA. The next block was randomized on the last evening or night of the current block, or during the first day of the next block. This procedure was repeated to produce four total consecutive blocks comprising eight three-day periods over a 24-day study period. The participant received a treatment-block assignment the same evening or night of the first night of that assignment. We designed the N1RT pilot with a small sample to keep our study feasible while allowing for basic assessment of our a priori hypotheses.

This treatment allocation scheme was chosen because we did not suspect a time trend in the outcomes (i.e., we assumed that neither blood glucose nor craving systematically increases or decreases over time). If there was no such time trend, then this scheme would enhance the generalizability (i.e., ecological validity) of our findings. For example, the participant would only able to anticipate within-block treatments, thereby discouraging them from changing their sleep behavior in anticipation of an upcoming treatment assignment (e.g., trying to “stock up” on sleep during the B period in anticipation of the A period in the next block). Hence, their observed sleep behavior may be more representative of their natural habits outside of the study in cases wherein a sleep-deprivation episode cannot be anticipated over a week in advance. However, if there was an outcome time trend, unobserved time-varying causes may have confounded the treatment effect for some randomized sequences (e.g., AB-AB-AB-AB or BA-BA-BA-BA). We would assess the veracity of this assumption in our main analyses.

### 2.5. Statistical Analysis Plan

We specified H1, H2, and H3 as follows, all with respect to the baseline condition and applicable to the effect sub-period. We specified H1 as the hypothesis that sleep deprivation causes higher mean BG level. We specified H2 as the hypothesis that sleep deprivation causes a higher probability of craving high-energy foods the following day. We specified H3 as the hypothesis that sleep deprivation causes a higher mean negative affect value the following day, as well as a lower mean positive value. We tested each hypothesis by modeling the mean outcome as a function of treatment, controlling for period and an interaction between treatment and period to account for a possible time trend over periods.

One key modeling assumption was that each model coefficient was identical across all time points after accounting for period. Another key assumption was that these residuals were statistically independent of each other. This latter assumption is supported if the residuals are only weakly or (better yet) not at all autocorrelated.

Hence, we assessed the veracity of these two assumptions as follows. For continuous outcomes, we tested for a time trend in residuals across time points. We tested for autocorrelation by checking the sample partial autocorrelation function (PACF) for the residuals (or summary of residuals, e.g., the mean) across whatever time increment was feasible to ensure evenly spaced time intervals between non-missing residual values. For binary outcomes, we tested whether time at a more granular level than period predicts the outcome probability more than the main-analysis period terms alone. We tested for autocorrelation by fitting a modified PACF that includes period, and that tested for associations of the current outcome with lagged outcomes.

All statistical hypothesis tests were conducted at the 0.05 significance level unless otherwise specified. Likewise, all confidence intervals provided 95% nominal coverage unless otherwise specified. All analyses were conducted in R (version 3.6.1). Because our main analytical objective was individualized, idiographic hypothesis refinement through exploratory analysis, we anticipated reporting a number of statistical findings based on post hoc hypotheses. That is, all statistical inferences were intended as first steps toward individualized refinement of our a priori hypotheses, rather than as evidence for or against established findings.

## 3. Results

Due to device failure after the first week, we were not able to collect the rest of EJD’s study data. We, therefore, only analyzed KW’s data. The 24-day study period for KW spanned 2–25 August 2017, with the first study night occurring on the night of 1 August 2017.

At the time of the experiment, KW was a healthy (i.e., no unusual medical history) 39-year-old female with normal BMI, who did not experience any notable work- or family-related stresses. Her prior sleep history was normal, with no self-reported sleep disturbances, sleep problems, or structural sleep apnea. KW described herself as a “good sleeper”; her sleep duration data for the three months prior indicate that she slept between 6 and 7 h most nights (see Figures S1 and S2). She did not have a sleeping partner or dependent and, therefore, was solely responsible for her own sleep, nutrition, and schedule. KW had normal menses, no menses during the trial, and self-reported no history of interaction between sleep quality and menstrual cycle phases [31,32]. Hence, we did not record whether or when she menstruated during the intervention period.

We checked for treatment compliance using Fitbit Charge 2™ sleep data (i.e., total sleep time in hours) that were collected for all nights. To achieve the assigned four hours on sleep-deprivation nights, KW stayed awake longer than usual on these nights, and set her alarm to wake up four hours later. The nights of sleep deprivation averaged 3.75 h with a standard deviation (SD) of 0.29; baseline nights averaged 7.20 (SD = 1.14) hours. These findings indicate good compliance with the instructed number of hours of sleep under both treatment conditions.

**Hypothesis** **1 (H1).** Effects of Sleep Deprivation on Blood Glucose.

*Descriptive summary.* There were 2601 recorded BG-level values. These values ranged from 39 to 254 mg/dL, with a mean and median of 77.8 and 72.0 mg/dL, respectively. BG levels were recorded 85 to 132 times each day, with a mean (and median) of 106 measurements per day. Of all measurements taken, 60 values (2.31%) were missing. BG levels were missing for 22 (2.54%) of 867 total measurements taken over all four effect sub-periods.

*Pre-processing.* Before the main analysis, we first tested whether the few missing values were missing at random (MAR) rather than missing completely at random (MCAR) [33,34] over the effect sub-periods as follows. We modeled the probability of observation (i.e., non-missingness) as a function of treatment, period, and their interaction through logistic regression. None of these predictors were statistically associated with missingness (i.e., non-intercept *p*-values from 0.715 to 0.909). Unless BG-level values were missing not at random (MNAR) [33,34], or if missingness was dependent on other predictors, these results supported the case that values were MCAR, and used only the 845 complete cases for our analyses. Hence, we concluded that BG-level values were MCAR. (An MNAR analysis was beyond the scope of this paper, as it would have involved making untestable assumptions, ideally supported by other studies, about the data-generating functions, and then conducting a sensitivity analysis by varying these assumptions.)

**Figure 1:**
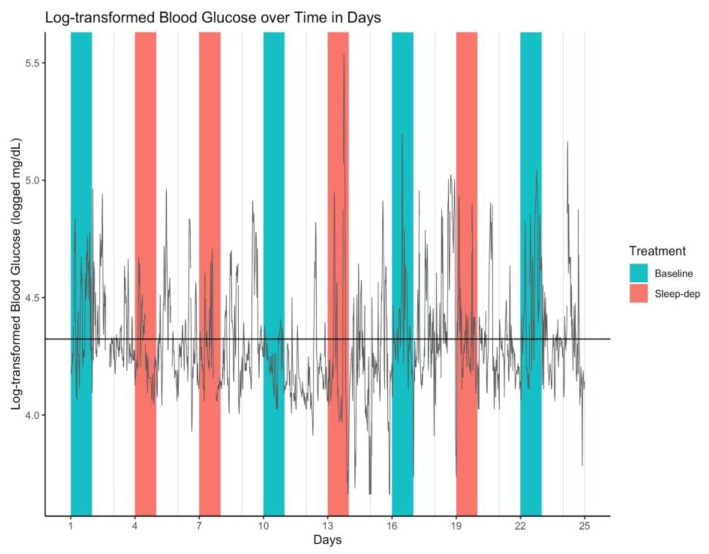
Log-transformed blood glucose (log-BG) time series. Gray line = time series of observed values; horizontal black line = empirical average log-BG value of 4.324; salmon/teal shaded regions = sleep-deprivation/baseline effect sub-period; vertical lines separate study days that start and end at 06:00.

Because BG levels were right-skewed in distribution, we log-transformed them, and used log-transformed BG (log-BG) in all analyses. The time series of all log-BG values is illustrated in Figure 1.

*Initial results.* Mean log-BG under baseline was estimated as 4.342 in period 1, with a confidence interval (CI) of 4.298 to 4.386. Sleep deprivation was estimated to decrease mean log-BG by 0.092 (CI: 0.026 to 0.159; *p* = 0.0063) while controlling for period, which was associated with an estimated 0.011 (CI: 0.001 to 0.021; *p* = 0.0230) mean increase in mean log-BG over each period. The latter finding indicated a slightly increasing time trend over periods. We also included an interaction term between treatment and period, but this was not statistically associated with the outcome (i.e., not statistically significant at *p* = 0.4392), suggesting that the effect of sleep deprivation on log-BG did not vary by period. Hence, we initially concluded that sleep deprivation slightly decreased mean BG level, even after accounting for a slight time trend across periods. Specifically, sleep deprivation proportionately reduced mean BG level by an estimated factor of 0.912 (CI: 0.853 to 0.974) (calculated by exponentiating the log-BG estimated coefficient).

*Temporal trend.* We tested for an overall temporal trend by modeling the residuals as a linear function of time. The residuals did not statistically change over time (*p* = 0.93). Hence, we concluded that while mean log-BG increased slightly over effect sub-periods, there was no overall time trend in mean log-BG after accounting for period.

*Autocorrelation.* We tested for autocorrelation by checking the sample PACF for the residuals as follows. Lags 1 and 2 were the most highly correlated with current log-BG level, with coefficient estimates 0.948 (*p* << 0.0001) and −0.493 (*p* << 0.0001), respectively. Other lags were statistically associated with current log-BG level, but had much weaker correlations, e.g., lag 3 estimate of 0.102 (*p* = 0.0034), lag 6 estimate of −0.071 (*p* = 0.044). Hence, we concluded that our original model could be re-specified by including the first two lags as predictors.

*Post hoc results.* We re-fit our H1 model on the original data as follows. We first tested whether missing values were MAR rather than MCAR with respect to the new set of predictors, i.e., treatment, period, their interaction, and the first two log-BG lagged values as model covariates (whenever they were observed). None of these predictors were statistically associated with missingness (i.e., non-intercept p-values from 0.668 to 0.975), again supporting our assumption that BG-level values were MCAR. In our main analysis, we found that sleep deprivation was no longer estimated to have had a mean effect on log-BG (*p* = 0.66). Period likewise was no longer associated with mean log-BG (*p* = 0.38). As before, the interaction term was not statistically associated with the outcome (*p* = 0.80). The two lagged outcomes, as expected, were statistically associated with log-BG; the coefficient estimates were 1.403 (CI: 1.342 to 1.464; *p* << 0.0001) and −0.486 (CI: −0.547 to −0.425; *p* << 0.0001). Hence, our post hoc H1 conclusion was that sleep deprivation did not change mean BG level, which itself did not change systematically across periods. That is, BG level was explained by its two consecutive past values more than by sleep deprivation or period.

**Hypothesis** **2 (H2).** Effects of Sleep Deprivation on Craving.

*Descriptive summary.* There were 60 recorded craving responses, with 40 (66.7%) “no” and 20 (33.3%) “yes” responses. The subject recorded responses in three days segments defined as early, midday, and late times throughout the day, respectively centered around 09:18, 14:58, and 22:43 (found using k-means clustering with three clusters specified). Craving responses were recorded 1–5 times each day, with a mean and median of 3.167 and 3.000 responses per day, respectively. Of 72 scheduled responses, 20 values (27.8%) were missing, i.e., early on days 4, 5, 7, 11, 12, 14, 17, 19, and 23, midday on days 2, 5, 6, and 9, and late on days 3, 4, 9, 11, 14, 17, and 22. Craving responses were missing for 5 (20.8%) of 24 total scheduled measurements over all four effect sub-periods.

*Pre-processing.* We first tested whether the five missing values were MAR rather than MCAR over the effect sub-periods as follows. We modeled the probability of observation as a function of treatment, period, and their interaction through logistic regression. None of these predictors were strongly statistically associated with missingness (i.e., *p* = 0.995 for all non-intercept predictors), supporting the assumption that craving responses were MCAR. Hence, we used only the 19 complete cases for our analyses.

Eight scheduled responses (11.1%) were entered twice at each of eight day segments, i.e., midday on days 4, 7, 17, 19, and 23, and late on days 2, 5, and 6. Both responses matched (i.e., both “yes” or “no”) on days 5, 6, 17, 19, and 23. The responses on days 2, 4, and 7 were all “yes” followed a little later by “no”. For our main analyses, we first used these earlier “yes” responses on days 2, 4, and 7, with a resulting total of 52 values used in our analyses, i.e., 33 (63.5%) “no” 19 (36.5%) “yes” responses. We then conducted a sensitivity analysis that used the later “no” responses on these days, with a resulting total of 36 (69.2%) “no” and 16 (30.8%) “yes” responses.

*Initial results.* The probability of craving under baseline was estimated as 0.333 (CI: 0.022 to 0.840) in period 1, with corresponding odds of 0.500 (CI: 0.023 to 5.221). Neither sleep deprivation, period, nor their interaction was associated with craving probability (*p* = 0.998, *p* = 0.999, *p* = 0.999, respectively). The sensitivity analysis yielded similar findings (*p* = 0.627, *p* = 0.997, *p* = 0.997, respectively). Hence, we initially concluded that sleep deprivation did not affect craving probability after accounting for period.

*Temporal trend.* We tested for a temporal trend by running the analysis with an additional term for time point. This additional term was not statistically associated with craving probability in either main or sensitivity analysis (*p* = 0.999 and *p* = 0.223, respectively). Hence, we concluded that there was no overall time trend in craving response after accounting for period.

*Autocorrelation.* We tested for autocorrelation by checking the modified sample PACF described earlier. The lag-1 craving responses were not statistically associated with current craving response for either main or sensitivity analysis (*p* = 0.998 and *p* = 0.996, respectively). Hence, we concluded that autocorrelation was negligible.

*Post hoc results.* Because we found no notable statistical association between period and craving probability, we fit a reduced model that included only treatment as a predictor. We first tested whether missing values were MAR rather than MCAR with respect to treatment alone. Treatment was not statistically associated with missingness (*p* = 0.159), again supporting our assumption that craving responses were MCAR.

Using this reduced model, we estimated the probability of craving under baseline as 0.091 (CI: 0.005 to 0.343), with corresponding odds of 0.100 (CI: 0.005 to 0.523). Sleep deprivation statistically increased the odds of craving by an estimated odds ratio of 30.000 (CI: 3.049 to 783.620; *p* = 0.0105). Under the sensitivity analysis, sleep deprivation also statistically increased the odds of craving, but only by an estimated odds ratio of 10.000 (CI: 1.074 to 232.306; *p* = 0.0687). This reduction in odds ratio estimate from that of the main analysis might reflect the subject giving into the earlier-reported craving, subsequently reporting not having a craving later within the same day segment, and thereby reducing the estimated effect of sleep deprivation when modeled using the later response.

Hence, our post hoc H2 conclusion based on both the main and sensitivity analyses was that sleep deprivation increased the odds of craving by an estimated odds ratio between 1.074 and 783.620. That is, we concluded that the participant was more likely to crave food after a night of sleep deprivation compared to baseline.

One explanation for this increase in statistical discernibility is that including period in the model reduced the effective sample size to assess craving probability to at most three observations in each of eight periods. Including the interaction term would have further reduced the effective sample size. Furthermore, sleep deprivation was estimated to have increased the odds of craving in the original full model (though, again, without strong statistical evidence). This estimate is at least consistent with sleep deprivation increasing craving.

**Hypothesis** **3 (H3).** Effects of Sleep Deprivation on Emotions.

*Descriptive summary.* There were 37 recorded values of both positive and negative scales. Positive values ranged from 19 to 49, with a mean and median of 38.2 and 38, respectively. Negative values ranged from 10 to 34, with a mean and median of 15.9 and 15, respectively. The subject recorded values at two days segments defined as early and late in the day, centered around 10:16 and 23:13 (found using k-means clustering with two clusters specified). For both positive and negative scales, PANAS values were recorded 0–2 times each day, with a mean and median of 1.54 and 2.00 measurements per day, respectively. Of 48 scheduled measurements, 11 values (22.9%) were missing; both positive and negative values were simultaneously observed or missing. No PANAS values were recorded on study days 11 and 14, both of which were the second day in each respective period (i.e., the first of the two washout days). PANAS values were missing for 2 (12.5%) of 16 total scheduled measurements (for each scale) over all four effect sub-periods.

**Figure 2:**
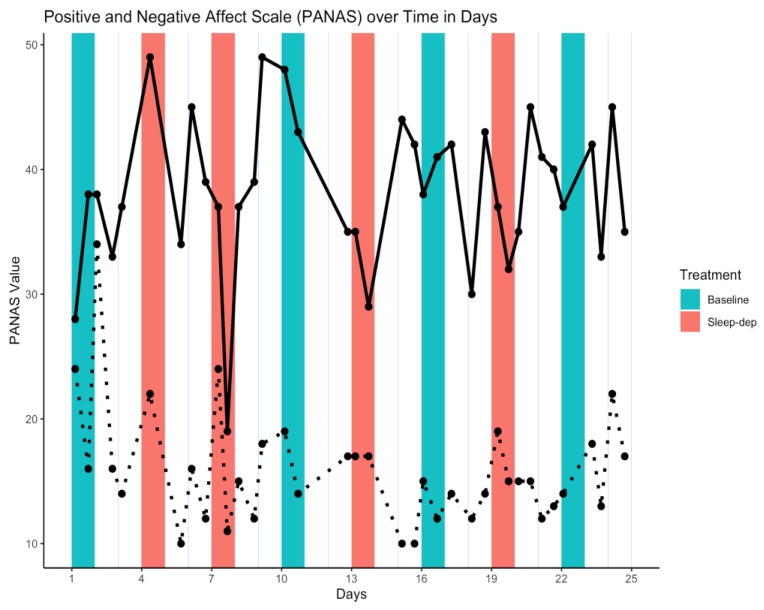
Positive and Negative Affect Scale (PANAS) time series. Solid/dotted line = time series of observed positive/negative values; salmon/teal shaded regions = sleep-deprivation/baseline effect sub-period; vertical lines separate study days that start and end at 06:00.

*Pre-processing.* We first tested whether the 11 missing values were MAR rather than MCAR over the effect sub-periods as follows. We modeled the probability of observation as a function of treatment, period, and their interaction through logistic regression. None of these predictors were strongly statistically associated with missingness (i.e., *p* = 0.997 at minimum for all non-intercept predictors), supporting the assumption that PANAS values were MCAR. Hence, we used only the 14 complete cases for our analyses. The time series of PANAS values is illustrated in Figure 2.

*Initial results.* Mean positive and negative values under baseline were estimated as 36.621 (CI: 26.29 to 46.95) and 19.662 (CI 14.72 to 24.607) in period 1, respectively. Sleep deprivation did not statistically decrease mean positive or negative values (*p* = 0.978 and *p* = 0.931, respectively) while controlling for period. Period was not statistically associated with mean positive values (*p* = 0.587), but there was some evidence for a slight decrease of 1.028 per period in mean negative values (*p* = 0.127). An interaction term between treatment and period was not statistically associated with either outcome (*p* = 0.492 and *p* = 0.691, respectively). Hence, we concluded that sleep deprivation did not affect mean PANAS values.

*Temporal trend.* We tested for an overall temporal trend by modeling the residuals as a linear function of time. The residuals did not statistically change over time for either positive or negative values (*p* = 0.98 and *p* = 0.92, respectively). Hence, we concluded that there was no overall time trend in PANAS values after accounting for period.

*Autocorrelation.* We tested for autocorrelation by checking the sample PACF for the lag-1 residuals as follows. Lagged positive-value residuals were not correlated with current positive values (*p* = 0.96). However, lagged negative-value residuals were slightly correlated with current negative values, with coefficient estimate −0.925 (*p* = 0.055). Hence, we concluded that autocorrelation was negligible for positive values, but that our original model for negative PANAS values could be re-specified by including the first lag as a predictor.

*Post hoc results.* Because we found no notable statistical association between period and negative PANAS values, we fit a reduced model that included only treatment as a predictor. We also included lag-1 negative values, as suggested by our autocorrelation analysis findings, and an interaction between treatment and lag-1 negative values.

We first tested whether missing values were MAR rather than MCAR with respect to the new set of predictors, i.e., treatment, the first negative lagged value, and their interaction as model covariates (whenever they were observed). None of these predictors were statistically associated with missingness (i.e., *p* = 0.998 for all non-intercept terms), again supporting our assumption that negative PANAS values were MCAR.

Using this model, we estimated the mean negative value under baseline as 5.443 (CI: 4.20 to 6.69). We also found that sleep deprivation statistically increased negative PANAS values by an estimate of 25.8 points (CI: 23.79 to 27.833; *p* = 0.0016) on average. Lagged negative values were statistically associated with an estimated 0.4426 (CI: 0.38 to 0.506; *p* = 0.0052) mean increase in current values under baseline, though this was negated by a 1.2888 (CI: 1.188 to 1.390; *p* = 0.0016) mean decrease in current values after a night of sleep deprivation. Hence, our post hoc H3 conclusion was that while sleep deprivation did not affect positive PANAS values, it increased negative PANAS values—though the effect was slightly dampened with larger previous negative values. That is, sleep deprivation increased negative emotions the most when previous negative emotions were low.

**Hypothesis** **1 Post Hoc.** Effects of Sleep Deprivation on Variability of Blood Glucose.

Because of the high variability in log-BG values seen in Figure 1, we hypothesized that there may be a smooth underlying or latent function of mean log-BG values, and that this function might vary by treatment group. That is, we interpreted loss of blood sugar control as a higher variability in log-BG values over time, and we specified this as a post hoc hypothesis.

One principled approach in characterizing such a smooth latent function that can be compared across treatments is to fit a mixed-effects model, wherein period is treated as a source of residual random-effect variation (e.g., through random intercept or slope terms) that is independent across effect sub-periods [3]. In our case, this approach is feasible because we found log-BG to be notably autocorrelated only up to two lags, implying that only the first two log-BG values per period are correlated with any values (in particular, the last two) from the previous period. Because washout sub-periods separate effect sub-periods by more than two time points, we can reasonably assume that log-BG values between effect sub-periods are independent. To account for within-period autocorrelation, a mixed-effect model specifies the autocorrelation as a function of random effect terms and time, rather than relying on explicit lag terms.

After some preliminary model exploration, we decided to report on a mixed-effects model that included treatment, six polynomial time-point terms, and the interactions between treatment and all time terms. We included random terms for the intercept and for time point (linear only; no polynomial terms). The strength of statistical evidence for determining whether and how a fixed-effect predictor was statistically associated with log-BG level was judged by examining the profiled 95% confidence intervals, i.e., if zero was not contained within the lower and upper bounds, we concluded there was strong statistical evidence of an association.

**Figure 3:**
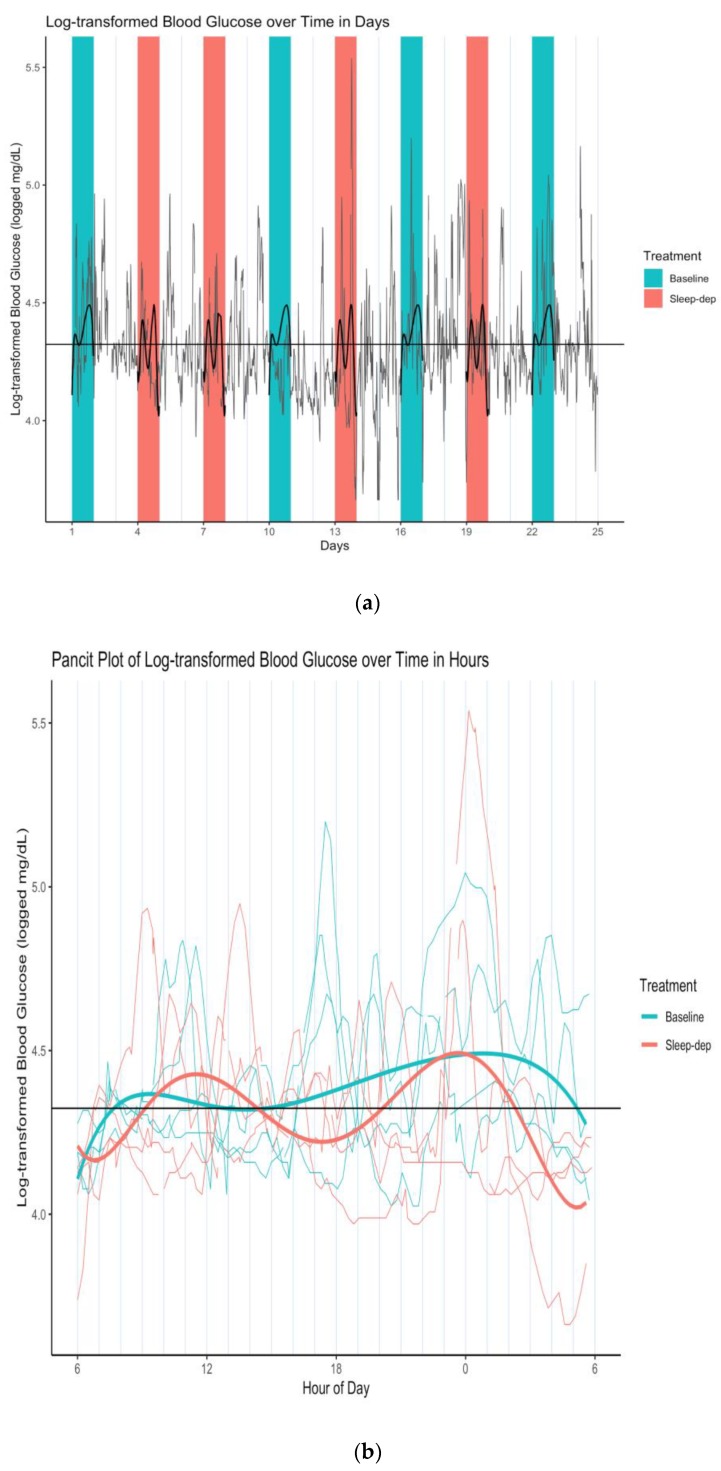
(**a**) Log-transformed blood glucose (log-BG) time series and latent mean log-BG curves estimated over effect sub-periods. Gray line = time series of observed values; horizontal black line = empirical average log-BG value of 4.324; black lines = estimated latent mean log-BG curves; salmon/teal shaded regions = sleep-deprivation/baseline effect sub-period; vertical lines separate study days that start and end at 06:00. (**b**) Log-transformed blood glucose (log-BG) pancit plot and latent mean log-BG curves estimated over effect sub-periods. Salmon/teal thin lines = time series of observed values for distinct effect sub-periods under sleep-deprivation/baseline; salmon/teal thick lines = estimated latent mean log-BG under sleep-deprivation/baseline; horizontal black line = empirical average log-BG value of 4.324; vertical lines separate hours.

We thereby concluded that sleep deprivation did not linearly affect log-BG levels, though there was a linear and squared time trend in log-BG levels at baseline (numerical results available in Supplementary Materials). However, the sleep-deprivation interactions with all time terms had statistically apparent associations with log-BG level, indicating that sleep deprivation may have increased the variability in latent mean log-BG levels over time. This is more easily seen in Figure 3a, which is a plot of Figure 1 overlaid with the latent curves of mean log-BG levels under each treatment condition estimated by our mixed-effects model. This visualization highlights how the two latent curves under baseline and sleep deprivation are similar in form, per our findings, but importantly that the sleep-deprivation curve has more amplified swings up and down (i.e., greater variability over time) when compared to baseline—possibly indicating greater volatility of latent mean log-BG over time.

This difference in time-dependent variability is particularly apparent in Figure 3b, which is the corresponding pancit plot. A pancit plot is a graph of partitioned time series segments that correspond to distinct treatment periods, and that are plotted together over the length of one period [3]. Such plots are directly analogous to spaghetti plots, graphs of time-dependent outcomes that correspond to different study individuals in a longitudinal analysis.

**Figure 4:**
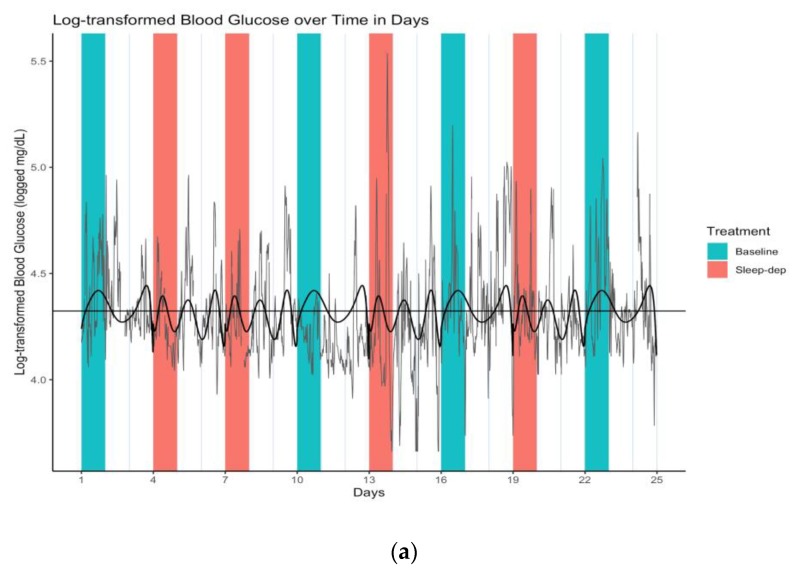

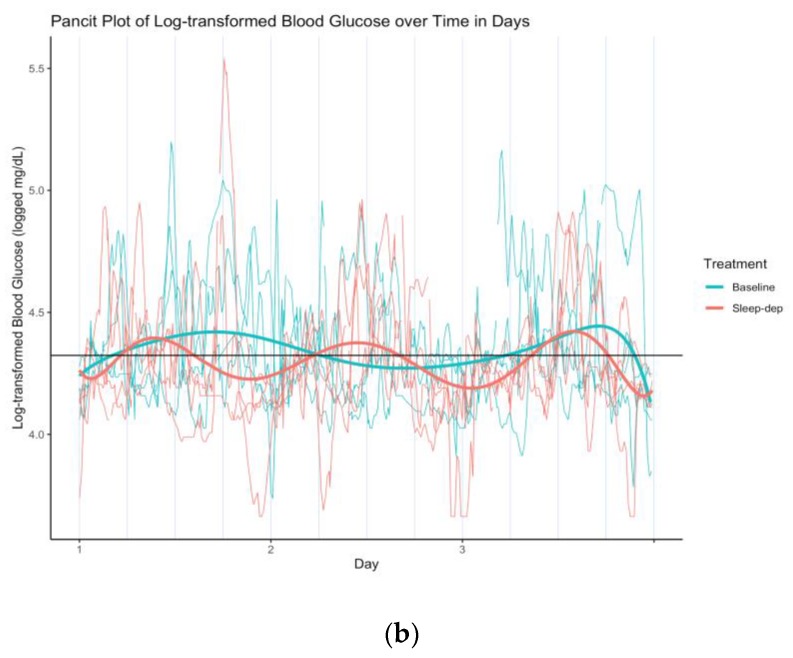
(**a**) Log-transformed blood glucose (log-BG) time series and latent mean log-BG curves estimated over effect and washout sub-periods. Gray line = time series of observed values; horizontal black line = empirical average log-BG value of 4.324; black lines = estimated latent mean log-BG curves; salmon/teal shaded regions = sleep-deprivation/baseline effect sub-period; vertical lines separate study days that start and end at 06:00. (**b**) Log-transformed blood glucose (log-BG) pancit plot and latent mean log-BG curves estimated over effect and washout sub-periods. Salmon/teal thin lines = time series of observed values for distinct periods under sleep-deprivation/baseline; salmon/teal thick lines = estimated latent mean log-BG under sleep-deprivation/baseline; horizontal black line = empirical average log-BG value of 4.324; vertical lines separate period days.

When we modeled latent mean log-BG level across entire periods (i.e., including both effect and washout sub-periods), the increase in time-dependent latent mean log-BG variability became even more apparent (numerical results available in Supplementary Materials). In this analysis, sleep deprivation also had no statistically apparent effect on log-BG levels, which did not statistically vary over time. However, as in the prior analysis conducted only over effect sub-periods, the sleep-deprivation interactions with all time terms had statistically apparent associations with log-BG level. This is illustrated in Figure 4a,b.

Both model findings were qualitatively consistent, and hence we concluded that sleep deprivation may have increased the variability in latent mean log-BG levels over time. Still, our mixed-effect model findings should be interpreted cautiously because both models assume that the residuals are normally distributed, which is largely true, except perhaps in the tails. In particular, Shapiro–Wilk tests of normality indicate possible non-normality (*p* << 0.0001). QQ-plots (not shown) show that this stems from non-normal behavior in the tails of the residuals.

## 4. Discussion

Our a priori hypotheses in general were not supported. Importantly, this study specifically challenged the individual’s own assumptions about herself, as well as the literature on sleep and our outcomes. While the individual verbally reported in a post-treatment interview that she believed she craved more sugar and felt more negative post-acute sleep deprivation, these personal assumptions were not supported in her data. Further, while research shows sleep deprivation can affect non-diabetic glucose levels, our a priori findings show that this generalization did not translate to this individual.

However, our post hoc findings provide targeted guidance for designing a follow-up idiographic study. While sleep deprivation did not affect BG levels, it may have increased the fluctuation of mean BG levels over time. Sleep deprivation may also have increased craving overall, regardless of treatment period. And sleep deprivation may have increased negative emotions, with this effect being most pronounced when negative emotions were low earlier that day or night.

## 5. Conclusions

An influx of self-tracking devices addresses the popular drive to quantify our own behaviors. But beyond self-interested knowledge, these devices and data, if used properly, can provide more rigorous insight into how individuals respond to various treatments and choices across multiple domains. Our n-of-1 trial showcases an elegant study design and statistical exploration of how sleep deprivation may affect blood glucose levels, craving, and affect in an individual when compared to regular sleep.

Both a priori and post hoc findings, together with the detailed processes (laid out in the Methods and Results sections) by which we arrived at them, more broadly inform n-of-1 trial designers of both what to look for in their own investigations and how to go about doing so. In a world where health treatments and behavior recommendations are based on population averages, n-of-1 trials and tracking devices open the possibilities for finding out how these recommendations actually affect their own body reactions to various changes.

Our study had the following limitations. We did not assess carryover of treatment effects from past periods, instead assuming the washout sub-periods eliminated such carryover. In a future study, we will check for possible carryover, and more fully characterize outcomes throughout an entire period (i.e., during both effect and washout sub-periods). A future study with both longer study periods and more frequently collected data would also allow us to flexibly model the dependence of each outcome on both treatment and various sets of predictors; for example, using a cross-validated random forest approach applied within the N1RT framework, following Daza (2019) [3]. The forthcoming CAPTEuR package in R facilitates such an analysis and also produces pancit plots and estimated average period treatment effect (APTE) trajectories, which are contrasts between mean trends (e.g., the difference between the two estimated latent curves in the Results section) [2,3]. Our mixed-effects modeling results also suggest that functional analysis could be used to better model a latent mean outcome.

In follow-up studies, we will characterize the idiographic effect of sleep deprivation on the following five outcomes, which we had also measured. We will assess the types of foods craved, defined as a food (specific) or food type (e.g., high fat, high salt, high sugar). We will assess craving fulfillment, defined as whether or not a participant would (or already did) give into the craving. Craved food and craving fulfillment were scheduled to be measured via self-report at the start, middle, and end of each 24-h study day, i.e., upon waking, during waking hours, and before going to bed, respectively. (The participant also recorded notes on behaviors, foods, or contexts of cravings as free text.) We will assess sleep quality the previous night, which was self-reported at the start of each 24-h study day using a 5-point Likert scale. We will assess diet healthfulness rating, which was measured throughout each 24-h study day, and later assessed and summarized by a registered dietitian and non-participant author (MO) using a 5-point Likert scale. Finally, we will examine sleep and physical activity as measured using actigraphy metrics produced by a Fitbit smartwatch, recorded passively and automatically throughout the study period.

## Supplementary Materials

The following are available online at https://www.mdpi.com/2227-9032/8/1/6/s1, Figure S1: Participant KW’s monthly Fitbit sleep duration data (i.e., total sleep time) for February through October 2017, Figure S2: Participant KW’s weekly Fitbit sleep duration data (i.e., total sleep time) for April through September 2017.
