# Supplementary material for: Effects of Sleep Deprivation on Blood Glucose, Food Cravings, and Affect in a Non-Diabetic: An N-of-1 Randomized Pilot Study
Source: Healthcare (Basel). 2019 Dec 25;8(1):6. doi: 10.3390/healthcare8010006 (PMC7151045; doi:10.3390/healthcare8010006)
Supplement: Supplementary file 1 [file healthcare-08-00006-s001.pdf]

healthcare

MDPI

Supplementary Materials

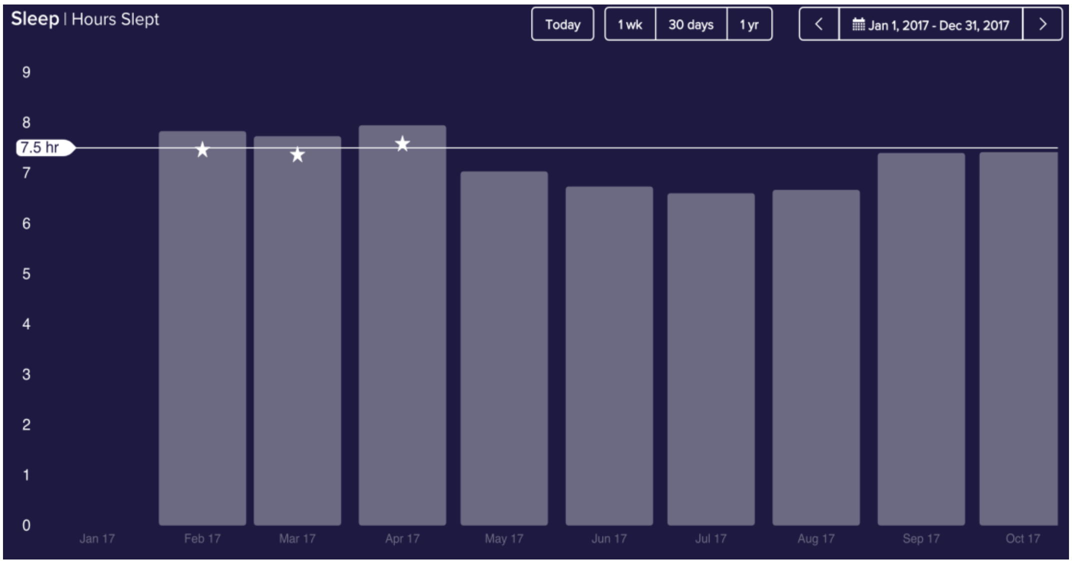

Figure S1. Participant KW's monthly Fitbit sleep duration data (i.e., total sleep time) for February through October 2017.

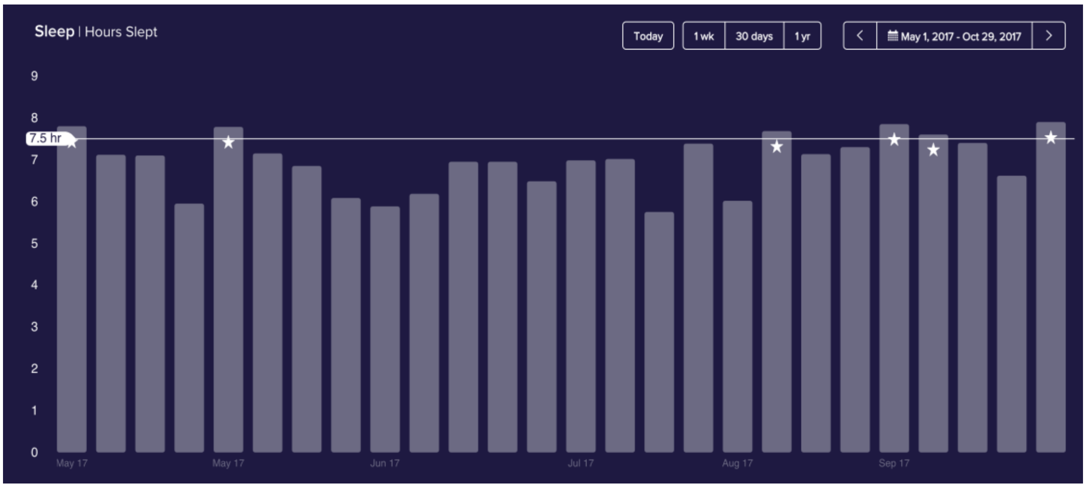

Figure S2. Participant KW's weekly Fitbit sleep duration data (i.e., total sleep time) for April through September 2017. (Note: The first "May 17" instance is an incorrect display artifact produced by the Fitbit app, and should read "Apr 17").

Mixed-Effects Model Numerical Results: Effect Sub-period Analysis

```
Linear mixed model fit by REML ['lmerMod']
Formula: log_bg.level ~ treatment * withinstudyday_timept + treatment *
    withinstudyday_timept2 + treatment * withinstudyday_timept3 +
    treatment * withinstudyday_timept4 + treatment * withinstudyday_timept5 +
    treatment * withinstudyday_timept6 + (withinstudyday_timept |      period)
   Data: tbl_bglong_h1

REML criterion at convergence: 140.1

Scaled residuals:
    Min      1Q  Median      3Q     Max
-2.2630 -0.6285 -0.0594  0.5258  4.5156

Random effects:
 Groups   Name                  Variance  Std.Dev. Corr
 period   (Intercept)           2.757e-03 0.052509
          withinstudyday_timept 6.748e-05 0.008215 -0.57
 Residual                       5.057e-02 0.224874
Number of obs: 845, groups:  period, 8

Fixed effects:
                                                   Estimate Std. Error t value
(Intercept)                                       4.108e+00  7.547e-02  54.431
treatment1. A (Sleep-Dep)                         1.011e-01  1.063e-01   0.950
withinstudyday_timept                             2.113e-01  8.315e-02   2.541
withinstudyday_timept2                           -6.113e-02  3.108e-02  -1.967
withinstudyday_timept3                            7.796e-03  4.987e-03   1.563
withinstudyday_timept4                           -4.907e-04  3.858e-04  -1.272
withinstudyday_timept5                            1.535e-05  1.420e-05   1.080
withinstudyday_timept6                           -1.932e-07  1.997e-07  -0.968
treatment1. A (Sleep-Dep):withinstudyday_timept  -3.353e-01  1.171e-01  -2.864
treatment1. A (Sleep-Dep):withinstudyday_timept2  1.630e-01  4.361e-02   3.738
treatment1. A (Sleep-Dep):withinstudyday_timept3 -2.914e-02  6.975e-03  -4.177
treatment1. A (Sleep-Dep):withinstudyday_timept4  2.354e-03  5.384e-04   4.372
treatment1. A (Sleep-Dep):withinstudyday_timept5 -8.771e-05  1.979e-05  -4.432
treatment1. A (Sleep-Dep):withinstudyday_timept6  1.225e-06  2.778e-07   4.410

Correlation matrix not shown by default, as p = 14 > 12.
Use print(x, correlation=TRUE)  or
    vcov(x)        if you need it

fit warnings:
Some predictor variables are on very different scales: consider rescaling
convergence code: 0
Model failed to converge with max|grad| = 0.0905077 (tol = 0.002, component 1)

                                                         2.5 %        97.5 %
.sig01                                            0.000000e+00  9.571942e-02
.sig02                                           -1.000000e+00  1.000000e+00
.sig03                                            3.754423e-03  1.314100e-02
.sigma                                            2.132119e-01  2.347651e-01
(Intercept)                                       3.964133e+00  4.251895e+00
treatment1. A (Sleep-Dep)                        -1.020303e-01  3.033915e-01
withinstudyday_timept                             4.936739e-02  3.736656e-01
withinstudyday_timept2                           -1.218133e-01 -5.655432e-04
withinstudyday_timept3                           -1.928377e-03  1.752756e-02
withinstudyday_timept4                           -1.243079e-03  2.620329e-04
withinstudyday_timept5                           -1.237936e-05  4.303775e-05
withinstudyday_timept6                           -5.823552e-07  1.965750e-07
treatment1. A (Sleep-Dep):withinstudyday_timept  -5.632297e-01 -1.066787e-01
treatment1. A (Sleep-Dep):withinstudyday_timept2  7.789274e-02  2.480090e-01
treatment1. A (Sleep-Dep):withinstudyday_timept3 -4.273291e-02 -1.552311e-02
treatment1. A (Sleep-Dep):withinstudyday_timept4  1.303030e-03  3.403138e-03
treatment1. A (Sleep-Dep):withinstudyday_timept5 -1.262644e-04 -4.906767e-05
treatment1. A (Sleep-Dep):withinstudyday_timept6  6.825085e-07  1.766118e-06
```

Mixed-Effects Model Numerical Results: Entire-Period Analysis

```
Linear mixed model fit by REML ['lmerMod']
Formula: log_bg.level ~ treatment * withinperiod_timept + treatment *
    withinperiod_timept2 + treatment * withinperiod_timept3 +
    treatment * withinperiod_timept4 + treatment * withinperiod_timept5 +
    treatment * withinperiod_timept6 + treatment * withinperiod_timept7 +      treatment *
withinperiod_timept8 + (1 | period)
   Data: tbl_bglong

REML criterion at convergence: -22.5

Scaled residuals:
    Min      1Q  Median      3Q     Max
-3.3228 -0.6053 -0.1579  0.4255  6.2283

Random effects:
 Groups   Name        Variance Std.Dev.
 period   (Intercept) 0.005615 0.07493
 Residual             0.046244 0.21504
Number of obs: 2541, groups:  period, 8

Fixed effects:
                                                 Estimate Std. Error t value
(Intercept)                                     4.240e+00  6.413e-02  66.117
treatment1. A (Sleep-Dep)                       2.267e-02  9.074e-02   0.250
withinperiod_timept                             3.106e-02  3.357e-02   0.925
withinperiod_timept2                           -4.005e-03  7.011e-03  -0.571
withinperiod_timept3                            4.310e-04  6.640e-04   0.649
withinperiod_timept4                           -2.703e-05  3.330e-05  -0.812
withinperiod_timept5                            9.069e-07  9.436e-07   0.961
withinperiod_timept6                           -1.648e-08  1.517e-08  -1.087
withinperiod_timept7                            1.542e-10  1.288e-10   1.197
withinperiod_timept8                           -5.842e-13  4.490e-13  -1.301
treatment1. A (Sleep-Dep):withinperiod_timept  -8.465e-02  4.737e-02  -1.787
treatment1. A (Sleep-Dep):withinperiod_timept2  2.923e-02  9.897e-03   2.953
treatment1. A (Sleep-Dep):withinperiod_timept3 -3.725e-03  9.370e-04  -3.975
treatment1. A (Sleep-Dep):withinperiod_timept4  2.221e-04  4.696e-05   4.730
treatment1. A (Sleep-Dep):withinperiod_timept5 -6.980e-06  1.329e-06  -5.250
treatment1. A (Sleep-Dep):withinperiod_timept6  1.193e-07  2.135e-08   5.591
treatment1. A (Sleep-Dep):withinperiod_timept7 -1.052e-09  1.811e-10  -5.805
treatment1. A (Sleep-Dep):withinperiod_timept8  3.739e-12  6.306e-13   5.930

Correlation matrix not shown by default, as p = 18 > 12.
Use print(x, correlation=TRUE)  or
    vcov(x)        if you need it

fit warnings:
Some predictor variables are on very different scales: consider rescaling

                                                       2.5 %        97.5 %
.sig01                                          4.132347e-02  1.172637e-01
.sigma                                          2.085950e-01  2.204065e-01
(Intercept)                                     4.118962e+00  4.361331e+00
treatment1. A (Sleep-Dep)                      -1.488223e-01  1.941211e-01
withinperiod_timept                            -3.453895e-02  9.670494e-02
withinperiod_timept2                           -1.771505e-02  9.691361e-03
withinperiod_timept3                           -8.658398e-04  1.729576e-03
withinperiod_timept4                           -9.215576e-05  3.800145e-05
withinperiod_timept5                           -9.358435e-07  2.752697e-06
withinperiod_timept6                           -4.614979e-08  1.313833e-08
withinperiod_timept7                           -9.738768e-11  4.062645e-10
withinperiod_timept8                           -1.462718e-12  2.926917e-13
treatment1. A (Sleep-Dep):withinperiod_timept  -1.772204e-01  7.957646e-03
treatment1. A (Sleep-Dep):withinperiod_timept2  9.882921e-03  4.856967e-02
treatment1. A (Sleep-Dep):withinperiod_timept3 -5.556351e-03 -1.893450e-03
treatment1. A (Sleep-Dep):withinperiod_timept4  1.303798e-04  3.139358e-04
treatment1. A (Sleep-Dep):withinperiod_timept5 -9.578899e-06 -4.382116e-06
treatment1. A (Sleep-Dep):withinperiod_timept6  7.764496e-08  1.610876e-07
treatment1. A (Sleep-Dep):withinperiod_timept7 -1.405709e-09 -6.976359e-10
treatment1. A (Sleep-Dep):withinperiod_timept8  2.507320e-12  4.972509e-12
```
